# The hidden duplication past of the plant pathogen *Phytophthora *and its consequences for infection

**DOI:** 10.1186/1471-2164-11-353

**Published:** 2010-06-03

**Authors:** Cindy Martens, Yves Van de Peer

**Affiliations:** 1 Department of Plant Systems Biology, VIB, Technologiepark 927, B-9052 Ghent, Belgium; 2 Bioinformatics and Evolutionary Genomics, Department of Molecular Genetics, Technologiepark 927, Ghent University, B-9052 Ghent, Belgium

## Abstract

**Background:**

Oomycetes of the genus *Phytophthora *are pathogens that infect a wide range of plant species. For dicot hosts such as tomato, potato and soybean, *Phytophthora *is even the most important pathogen. Previous analyses of *Phytophthora *genomes uncovered many genes, large gene families and large genome sizes that can partially be explained by significant repeat expansion patterns.

**Results:**

Analysis of the complete genomes of three different *Phytophthora *species, using a newly developed approach, unveiled a large number of small duplicated blocks, mainly consisting of two or three consecutive genes. Further analysis of these duplicated genes and comparison with the known gene and genome duplication history of ten other eukaryotes including parasites, algae, plants, fungi, vertebrates and invertebrates, suggests that the ancestor of *P. infestans*, *P. sojae *and *P. ramorum *most likely underwent a whole genome duplication (WGD). Genes that have survived in duplicate are mainly genes that are known to be preferentially retained following WGDs, but also genes important for pathogenicity and infection of the different hosts seem to have been retained in excess. As a result, the WGD might have contributed to the evolutionary and pathogenic success of *Phytophthora*.

**Conclusions:**

The fact that we find many small blocks of duplicated genes indicates that the genomes of *Phytophthora *species have been heavily rearranged following the WGD. Most likely, the high repeat content in these genomes have played an important role in this rearrangement process. As a consequence, the paucity of retained larger duplicated blocks has greatly complicated previous attempts to detect remnants of a large-scale duplication event in *Phytophthora*. However, as we show here, our newly developed strategy to identify very small duplicated blocks might be a useful approach to uncover ancient polyploidy events, in particular for heavily rearranged genomes.

## Background

Oomycetes or water molds form a diverse group of eukaryotic micro-organisms that have originally been classified as Fungi because of their similarity in growth morphology, propagation through spores and weaponry to infect host organisms [1]. Furthermore, they occupy similar ecological niches and share many cell wall degrading enzymes to weaken host tissues [2,3]. However, biochemical and molecular data have shown that oomycetes have little affinity with "true" fungi but are instead more closely related to heterokont algae and diatoms [4,5], belonging to the assemblage chromalveolates, which also include organisms such as ciliates, apicomplexans and dinoflagellates [6,7]. Also in contrast to fungi, oomycetes are diploid organisms that lack a free haploid life stage.

Members of the genus *Phytophthora *cause devastating diseases on a wide range of plants, and are the most important pathogens of dicots. For instance, *Phytophthora infestans*, responsible for severe damage in the food production worldwide by infecting tomato and potato [8], was the infective agent of the so-called potato blight that caused the Irish famine between 1845 and 1849, during which approximately one million people died and another million emigrated [9,10]. Another species, *P. sojae*, causes root and stem rot in soybean resulting in huge annual production losses [11].

So far, three *Phytophthora *species have been fully sequenced and annotated, namely *P. sojae*, *P. ramorum and P. infestans*. Breakouts of the 'sudden oak death' disease caused by *P. ramorum *led to the first *Phytophthora *genome project. Since there were no close relatives sequenced yet, a second genome, the one of *Phytophthora sojae*, was sequenced simultaneously. *P. sojae *and *P. ramorum *have a genome size of 95 Mb and 65 Mb, respectively [12]. *P. infestans*, of which the genome sequence has been determined recently as well, has an estimated genome size of 240 Mb [13]. In comparison to other plant pathogens, the *Phytophthora *genomes are quite large. Bacterial genomes are often smaller than 10 Mb and fungal genomes rarely exceed 40 Mb [14]. The larger size of the *P. sojae *genome compared to *P. ramorum *is not only because of the higher number of predicted genes (16.988 and 14.451, respectively [13]) but also because of larger intergenic regions and different retrotransposon expansion patterns [12,15]. In *P. infestans*, which has 17.797 predicted genes [13], the intergenic regions are even larger than in *P. sojae *and the number of different types of transposons is overwhelming [13,16-18]. The *P. infestans *genome is by far the largest chromalveolate genome sequenced and Haas and colleagues (2009) have shown that its expansion results from a proliferation of repetitive DNA accounting for ~74% of the genome [13]. Comparison of the three *Phytophthora *genomes also revealed an unusual genome organization; i.e. regions with conserved gene order, high gene density and lower repeat content are separated by regions with non-conserved gene order, low gene density and high repeat content [13].

In a previous study, we observed that *Phytophthora *species have many more genes than most other chromalveolate species for which the complete genome sequence has been determined [19]. Also the average gene family size is larger than for the other chromalveolates, except for the ciliates *Paramecium tetraurelia*, which has undergone three whole genome duplication events [20] and *Tetrahymena thermophila*, which has undergone an extensive number of tandem duplications [21]. Furthermore, in particular genes important for the interaction with their hosts, such as genes encoding cell wall degrading enzymes, often seem to have been duplicated in *Phytophthora *species [19,22]. Here, we have tried to unravel the duplication past of the three *Phytophthora *species and conclude that many of the duplicated genes are likely the result of a shared ancient large-scale or even whole genome duplication event.

## Results

### Duplicated regions in the *Phytophthora *genome

If an organism has undergone a large-scale or whole genome duplication (WGD) in its evolutionary past, there is a reasonable chance to find remnants of this event. For instance, such remnants can be detected by the identification of genomic segments sharing a set of homologous genes [23]. When also the order of the homologous genes (sometimes referred to as anchor points or anchors) on the chromosomes is still conserved, the evidence for a block duplication is strengthened. To define homologous gene pairs within each of the *Phytophthora *species and reference organisms, the proteomes were grouped into gene families based on sequence similarity and Markov clustering (see Methods). Gene families with more than 100 members were omitted from the analysis since these gene families are often artefacts of the gene family clustering methods, i.e. artificial clustering of different families into superfamilies. Also gene pairs with a K_S_-value lower than 0.1 and/or lying on a small scaffold (i.e. fewer than 6 genes) were omitted from the analysis (see Methods).

Using our previously developed software i-ADHoRe [24], we identified blocks of homologous genes in the *Phytophthora *genomes. In brief, the i-ADHoRe algorithm detects homologous (duplicated) regions in a genome by identifying diagonals in a gene homology matrix, after which the longest diagonal or duplicated region is reported. The whole procedure is controlled by a set of parameters including gap size, which describes the maximal number of intervening, non-homologous genes tolerated between two homologous genes within a collinear segment, and a parameter determining to what extent the elements of a cluster fit on a diagonal line. Because of its specific development and implementation, the algorithm can only detect clusters of at least three homologous gene pairs [23,25].

To our surprise, the large majority of duplicated blocks in *P. infestans *consist of only three homologous gene pairs (only one block of five duplicated genes and seven blocks of four genes could be detected; data not shown). The same is observed for both other *Phytophthora *species, namely *P. sojae *and *P. ramorum *(data not shown). For all duplicated blocks, we also counted the number of intervening (non-homologous) genes. Strikingly, the average number of intervening genes is extremely small and in most cases the duplicated genes in these small blocks are located directly next to each other.

The fact that *Phytophthora *species, especially *P. infestans *and *P. sojae*, have a large number of genes and many multicopy gene families [19], as well as many duplicated blocks of three homologs, raised the question whether these blocks could be the remnants of a large-scale gene or even entire genome duplication event. Furthermore, the presence of a high number of very small duplicated blocks could point to an ancient duplication event followed by a large number of genome rearrangements breaking up larger blocks. If this were true, we would expect to find even more blocks with only two homologous genes.

### Finding small duplicated blocks of genes

**Figure 1:**
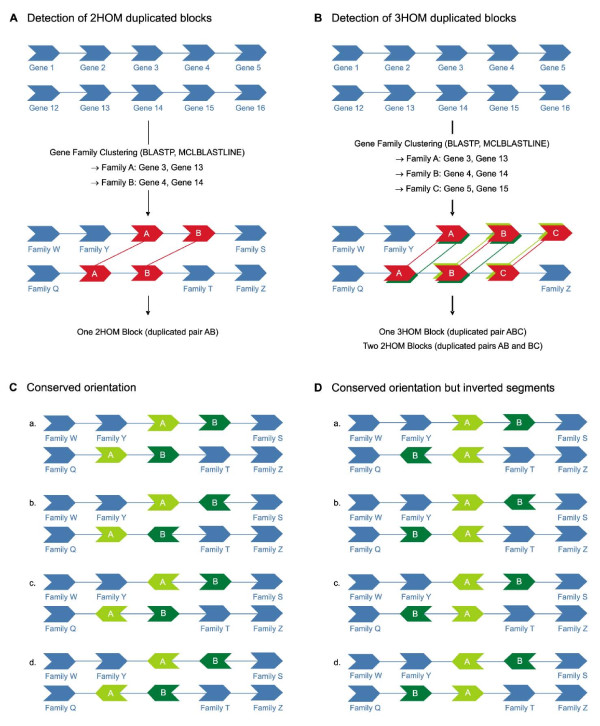
**Detection of small duplicated genomic segments**. **(A) **Detection of 2HOM blocks in real and random genomes. In red, a duplicated block of two homologous genes AB is shown. **(B) **Detection of 3HOM blocks in real and random genomes. In red, a duplicated block of three homologous genes ABC is shown. In this example, also two 2HOM blocks are identified, namely AB (dark green) and BC (light green). **(C) **All possible situations where the gene orientation in a 2HOM block is conserved and not inverted. **(D) **Possible situations where the gene orientation in a 2HOM block is conserved but where one segment is inverted.

Since i-ADHoRe and most other methods for duplicated block detection have been developed specifically to report large homologous segments between or within genomes [23], we developed a new approach to detect small duplicated blocks of only two or three homologous genes, referred to as 2HOM and 3HOM blocks, respectively (see Methods and Figure 1, Panel A and B). As can be seen in Figure 1 (Panel B), 2HOM blocks can be part of a 3HOM block, which is different from for example i-ADHoRe or related software reporting only the longest blocks. Similarly, our 3HOM blocks can be part of a block with more than three homologous genes. A genome that has been recently duplicated and did not undergo extensive genome rearrangements will have almost no blocks of only two or three anchor points using the i-ADHoRe approach because most of them will be part of a larger block. However, in our new strategy such a genome will count numerous 2HOM and 3HOM blocks, indicative of a large-scale duplication event. In this respect, we can compare the distribution of 2HOM and 3HOM blocks between duplicated and non-duplicated genomes, trying to reduce the effect of genome rearrangements and the breaking up of larger duplicated blocks. It should be noted that blocks comprised of gene families with more than 100 members were discarded from all further analyses.

**Figure 2:**
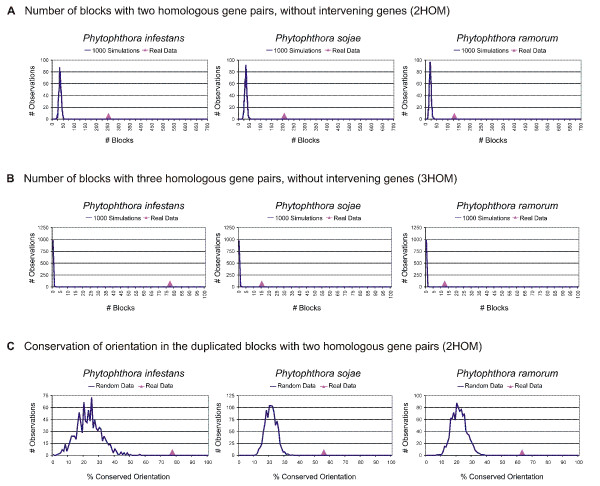
**Duplicated blocks and orientation analysis in *Phytophthora *and random genomes**. The number of **(A) **2HOM blocks and **(B) **3HOM blocks in *P. infestans*, *P. sojae*, and *P. ramorum *(pink triangles) versus the number of 2HOM, resp. 3HOM blocks in random genomes, i.e. the expected number of blocks based on 1000 simulations (blue distribution). **(C) **Orientation conservation in the 2HOM and 3HOM blocks in real versus random data.

Because we are looking at very small duplicated blocks, the possibility of finding some of those by chance is greater than for larger blocks. Therefore, in our block detection method, we have chosen to be more stringent by not allowing any intervening genes. Moreover, to compare our observation with what could be expected by chance, we ran 1000 simulations for every genome, i.e. every genome was shuffled 1000 times and for each random genome the number of 2HOM and 3HOM blocks was counted. The results for the 2HOM block detection in the three different *Phytophthora *species are shown in Figure 2A, where the pink triangle shows the number of blocks found in the real genome, while the distributions shown in blue represent the results for random data, i.e. the output of the 1000 simulations. If the number of blocks found in the real data is clearly different from the number of blocks found in the randomizations, the number of blocks in this species is highly significant, meaning that we find more blocks than we would expect by chance alone. As can be seen in Figure 2A, the number of 2HOM blocks detected in all three *Phytophthora *species is significantly different from what would be expected by chance alone. The same is true for the 3HOM blocks (Figure 2B), where we can also see that 3HOM blocks without intervening genes are extremely rare in randomized genomes. Moreover, as expected, in all three *Phytophthora *genomes the number of 2HOM blocks is much higher than the number of 3HOM blocks (Table 1).

**Table 1:** 2HOM and 3HOM block detection in *Phytophthora *and reference genomes.

Organism	No. of filtered blocks	No. of possible filtered blocks	Percentage of filtered blocks
**2HOM block detection**			
*P. infestans*	253	11,661	2.17
*P. sojae*	207	10,816	1.91
*P. ramorum*	128	8,706	1.47
*P. tricornutum*	24	9,559	0.25
*P. falciparum*	18	4,945	0.36
*S. cerevisiae*	51	5,516	0.92
*K. lactis*	12	5,235	0.23
*A. thaliana*	651	18,805	3.46
*H. sapiens*	258	16,746	1.54
*T. nigroviridis*	531	15,094	3.52
*C. elegans*	155	16,224	0.96
*D. melanogaster*	6	12,262	0.05
*A. gambiae*	46	10,140	0.45

**3HOM block detection**			
*P. infestans*	77	11,224	0.69
*P. sojae*	15	10,062	0.15
*P. ramorum*	12	8,206	0.15
*P. tricornutum*	2	9,525	0.02
*P. falciparum*	4	4,919	0.08 08
*S. cerevisiae*	8	5,516	0.15
*K. lactis*	5	5,233	0.10
*A. thaliana*	90	17,410	0.52
*H. sapiens*	28	15,868	0.18
*T. nigroviridis*	71	14,367	0.49
*C. elegans*	25	15,835	0.16
*D. melanogaster*	1	11,867	0.01
*A. gambiae*	2	9,898	0.02

Because the number of small duplicated blocks in *Phytophthora *genomes seems unexpectedly high, we compared them with the number of blocks found in other genomes, which we will further refer to as the reference genomes. We distinguished three types of reference genomes, i.e. those of organisms that (i) underwent at least one WGD in their evolutionary past (*Arabidopsis thaliana *[26,27], *Saccharomyces cerevisiae *[28,29], *Homo sapiens *[30], and *T. nigroviridis *[31,32]), (ii) underwent segmental duplications (*C. elegans *[33-36], *P. falciparum *[37] and *K. lactis *[38,39]), (iii) most likely have not been duplicated (*P. tricornutum*, *D. melanogaster *and *A. gambiae*). For all organisms, we applied the same detection strategy. The results for the detection of 2HOM and 3HOM blocks are shown in Table 1.

**Figure 3:**
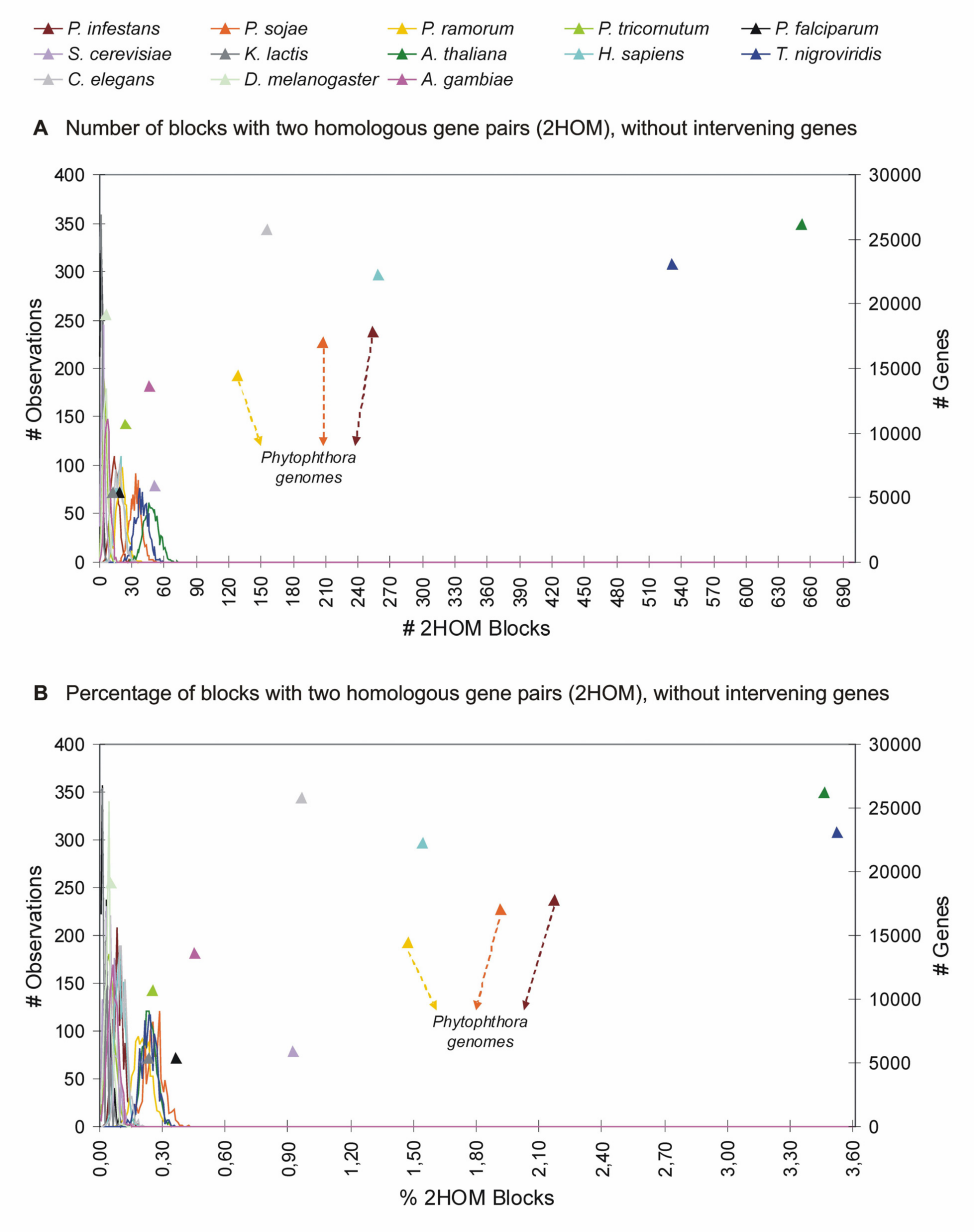
**Number (A) and percentage (B) of 2HOM blocks in the *Phytophthora *species and reference genomes**. The results for real genomes are represented by triangles, the results for the corresponding randomized genomes are represented by the distributions in the same color as the triangle data points. The left Y-axis applies to the random data, the right Y-axis applies to the real data.

Figure 3A shows the results of the 2HOM block simulation analyses for all genomes (for a detailed overview, see Additional file 1). Again, the colored triangles represent the number of blocks that we find in the real genomes, while the corresponding distributions represent the 1000 simulations for every genome. As can be observed, there are three clusters of organisms that can be discerned: one formed by the reference species that have not undergone large-scale gene duplication events, one by the three *Phytophthora *species, human and *C. elegans*, and one by *Arabidopsis *and *Tetraodon*. These latter two organisms show an extremely high number of 2HOM blocks, which is explained by the fact that both *Arabidopsis *and *Tetraodon *have undergone multiple rounds of WGDs. For instance, it has been shown recently that the Arabidopsis genome has undergone at least three WGD events, two of which have occurred during the last 70 or so my [26,27]. Ray-finned fishes, such as *Tetraodon*, have undergone a WGD about 300 mya, after their divergence from the land vertebrates [31,32]. On top of that, they share two rounds of earlier genome duplications with the other vertebrates [40]. So, all organisms for which it has been clearly demonstrated that they have doubled their genome during their evolutionary past show a very high number of 2HOM duplicated blocks. The only exception is the yeast *S. cerevisiae*, which has undergone a WGD 100 mya [28,31]. However, when we correct for the number of genes in the genome, this discrepancy disappears (see further).

Table 1 summarizes the results of the detection of the 3HOM blocks in the *Phytophthora *and reference genomes (for a detailed overview, see Additional file 2). Next to *A. thaliana*, *P. infestans *has the highest number of 3HOM blocks. The number of 3HOM blocks in the other two *Phytophthora *species is much lower, but still higher than in *S. cerevisiae*.

There is no evidence that the genome of *C. elegans *has been duplicated. However, it has been shown that this genome has undergone segmental duplication [33-36], which explains its relatively large number of 2- and 3HOM blocks. To investigate whether many small blocks in the *Phytophthora *genomes could also be explained by a few segmental or chromosomal duplications, we calculated the percentage of genomic scaffolds containing at least one small duplicated block. After removal of large gene families, 66, 67 and 61% of the *P. infestans*, *P. sojae *and *P. ramorum *scaffolds, respectively, contain at least one block. When we count the number of scaffolds with two up to 60 duplicated blocks, the number of scaffolds in all three *Phytophthora *species gradually decreases when the number of detected blocks increases (see Additional file 3). Moreover, we observed that the number of blocks detected on a scaffold is linearly correlated with the size of the scaffold, expressed in the number of genes (see Additional file 4). Finally, in order to make sure that the small duplicated blocks are not operon-like structures, we considered functional clustering and intergenic distances within the duplicated blocks (see Additional file 5). The results of these analyses rejected the operon-hypothesis (see Additional file 5 and Additional file 6).

### Dating the block duplications

The identification of many segmental duplications is usually considered strong evidence for a WGD, although it is hard to rule out that they are the result of many independent segmental duplications. However, if one can show that most gene duplicates have been created at about the same time, this provides additional evidence for a single duplication event [23]. Therefore, we have tried to date the *Phytophthora *paranomes based on third codon or synonymous substitution rates (or Ks-estimation, see Methods). Because most substitutions in third-codon positions do not result in amino-acid replacements, the rate of fixation of these substitutions is expected to be relatively constant in different protein-coding genes [41] and, therefore, to reflect the overall mutation rate [42].

**Figure 4:**
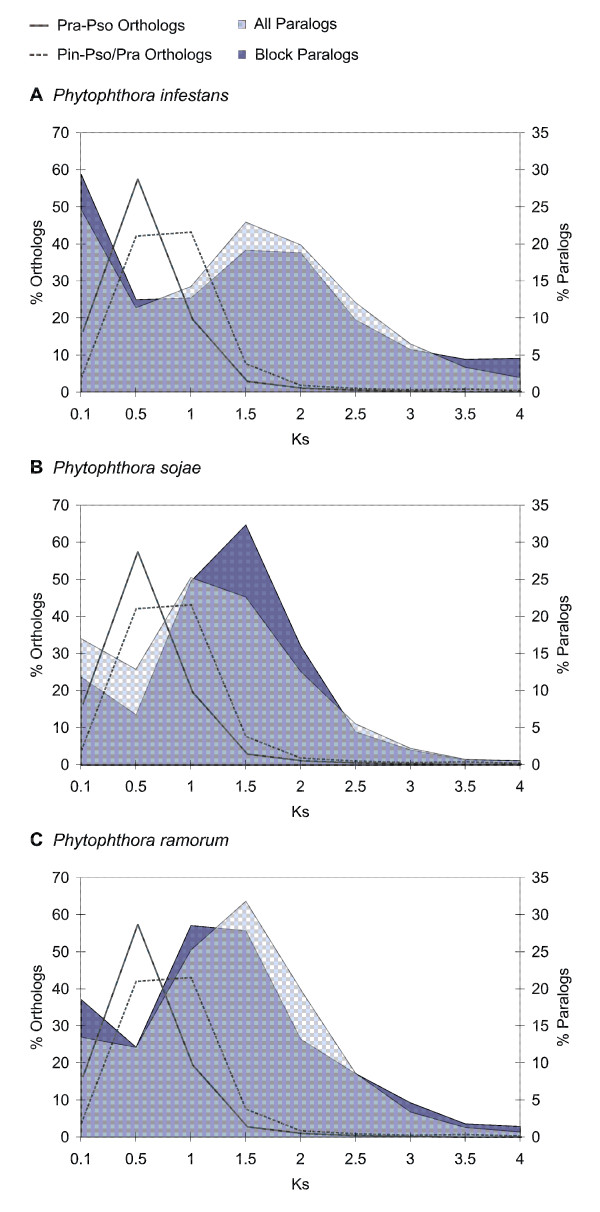
**K**_**S **_**dating of the *Phytophthora *duplication and speciation events**. K_S _distribution for paralogous and orthologous genes in the **(A) ***P. infestans*, **(B) ***P. sojae *and **(C) ***P. ramorum *genome. The K_S _distribution for the *P. sojae *(Pso) and *P. ramorum *(Pra) orthologs (left Y-axis), representing the *P. sojae*-*P. ramorum *speciation, is indicated by a full grey line. The K_S _distribution for the *P. sojae *(or *P. ramorum*) and *P. infestans *(Pin) orthologs (left Y-axis), representing the *P. infestans*-P. sojae/*P. ramorum *speciation, is indicated by a grey dotted line. The K_S _distribution of the whole paranome (right Y-axis) is shown in light blue shading; the K_S _distribution of the paralogs located in duplicated blocks (right Y-axis) is indicated in dark blue shading.

As can be seen in Figure 4 (grey line), the most recent speciation event is the divergence between *P. ramorum *and *P. sojae*. Prior to this event, *P. infestans *diverged from the common ancestor of *P. sojae *and *P. ramorum *(grey dotted line), which is in accordance with phylogenetic data [43]. Because of the fact that we only find small duplicated blocks, possibly because of many genome rearrangements (see further), many paralogs that arose through this large-scale duplication event will no longer lie in duplicated blocks. Therefore, we not only dated the homologous gene pairs still residing in duplicated blocks (Figure 4, dark blue shading), but also dated the whole paranome of the three species (Figure 4, light blue shading). As shown in Figure 4, both distributions have the same shape (it should be noted that the absolute values of the 'paranome' distribution are of course much higher). It is clear that in all three species there is a peak around K_S_-values of 1.5-2.0. Although we are aware that higher values of K_S _should be interpreted with caution due to saturation effects, it is clear that many paralogs arose around the same time in the three species and prior to two speciation events, and must thus have occurred in their common ancestor. This is also confirmed by the fact that *P. sojae *and *P. ramorum *still share 24 2HOM blocks (i.e., 11.6% resp. 18.8% of the total number of *P. sojae *resp. *P. ramorum *2HOM blocks). *P. sojae *and *P. ramorum *also share 19 (9.2%) resp. 11 (or 8.6%) of their 2HOM blocks with *P. infestans*.

### Gene orientation conservation in the duplicated blocks

We also studied gene orientation conservation in the *Phytophthora *and reference genomes. To this end, we have applied the strategy shown in Figure 1 (Panel C and D). In Panel C, the possible situations are shown where the orientation of the genes in 2HOM blocks is completely conserved. Since it is possible that during a WGD whole regions are being inverted, the different possibilities shown in Panel D are also considered conserved. All the other cases, where only one gene is inverted and the other one not etc., we define as the orientation not to be conserved. We have applied this strategy to all the genomes in our dataset and again ran 1000 simulations for every species. As can be seen in Figure 2C, 77% of the 2HOM blocks in *P. infestans *have a conserved gene orientation (pink triangle), whereas the conserved orientation in the reshuffled genomes is much smaller (blue line). In *P. sojae *and *P. ramorum *the conservation percentage is slightly lower, but still higher than in random data. In *A. thaliana*, *T. nigroviridis *and *H. sapiens*, again the situation is similar as in *Phytophthora*, although in human and *Tetraodon *the percentages are a bit higher (see Additional file 7). In *C. elegans *and *D. melanogaster *the conservation percentages are just below 50 and at the tail of the simulation curve. Also in *A. gambiae *the conservation percentage is at the tail of the random curve. For the other genomes it is difficult to conclude anything because there are too few data points. This is also the reason why this simulation analysis was not performed on the 3HOM blocks.

The fact that the blocks in the *Phytophthora *species have a conserved orientation provides further support for the homologous gene pairs to have been duplicated in concert. If the homologous gene pairs would have been duplicated separately and afterwards assembled into gene clusters for example, then the genes within the block could have easily been inverted, resulting in a non-conserved gene orientation within the block. Also Cavalcanti and colleagues showed that in yeast the number of blocks with the same gene order was similar to the number of blocks with the same gene order and gene orientation, while in *C. elegans *the number of blocks dropped substantially after imposing the orientation criterion [44].

### Gene retention following duplication in *Phytophthora*

Besides block duplicates, also the number of tandem duplicates is large in the *Phytophthora *genomes. In pathogenic species, it is well-known that genes related to pathogenesis are often located in tandem arrays (for example [19,45-47]. In a previous study, this was for example observed for *P. falciparum*, but also for both *P. sojae *and *P. ramorum *[19]. To see whether there is a similar bias in gene function for genes retained in the 2- and 3HOM blocks, we calculated GO Slim enrichments (see Methods) for both the tandem and block duplicate datasets. Table 2 shows the GO Slim labels that are significantly (Q-value < 0.05) enriched in (i) tandem duplicates, (ii) block duplicates, and (iii) in both tandem and duplicates.

**Table 2:** Significantly enriched GO-labels in the block and/or tandem duplicates of the *Phytophthora *species.

GO-tree	GO-label	GO-description	No. of TD*	No. of BD*	Relative to the total No. of TD and BD		Relative to the total No. of genes	
					
					% TD	% BD	% TD	% BD
**Only in tandem duplicates**								

P	GO:0006464	protein modification process	173	18	14.9	5.4	9.4	1.0

P	GO:0044403	symbiosis, encompassing mutualism through parasitism	28	4	2.4	1.2	25.0	3.6

P	GO:0006091	generation of precursor metabolites and energy	97	22	8.4	6.6	10.8	2.4

F	GO:0016301	kinase activity	163	15	10.4	3.7	10.5	1.0

F	GO:0008289	lipid binding	49	7	3.1	1.7	8.9	1.3

F	GO:0004672	protein kinase activity	155	15	9.9	3.7	12.2	1.2

C	GO:0005576	extracellular region	56	10	10.2	5.2	21.5	0.4

C	GO:0005764	lysosome	12	3	2.2	1.5	37.5	9.4

C	GO:0005773	vacuole	12	3	2.2	1.5	31.6	7.9

C	GO:0030312	external encapsulating structure	8	3	1.5	1.5	20.5	7.7

C	GO:0005618	cell wall	7	3	1.3	1.5	20	8.6

**Only in block duplicates**								

P	GO:0006996	organelle organization and biogenesis	45	26	3.9	7.8	5.8	3.3

P	GO:0007154	cell communication	53	24	4.6	7.2	5.9	2.7

P	GO:0007165	signal transduction	19	22	1.6	6.6	3.2	3.7

P	GO:0015031	protein transport	4	12	0.3	3.6	1.0	3.0

P	GO:0016043	cellular component organization and biogenesis	59	45	5.1	13.5	4.5	3.4

F	GO:0003779	actin binding	8	8	0.5	2.0	9.1	9.1

F	GO:0004871	signal transducer activity	10	15	0.6	3.7	4.0	5.9

F	GO:0004872	receptor activity	10	14	0.6	3.5	7.4	10.3

F	GO:0005509	calcium ion binding	13	19	0.8	4.7	2.1	3.0

F	GO:0008092	cytoskeletal protein binding	8	8	0.5	2.0	8.1	8.1

F	GO:0030528	transcription regulator activity	29	12	1.9	3.0	7.0	2.9

**In tandem and block duplicates**								

P	GO:0005975	carbohydrate metabolic process	174	39	15.0	11.7	19.2	4.3

P	GO:0006810	transport	326	120	28.1	35.9	10.5	3.9

P	GO:0006811	ion transport	53	29	4.6	8.7	9.0	4.9

P	GO:0006950	response to stress	53	20	4.6	6.0	12.0	4.5

P	GO:0009605	response to external stimulus	10	8	0.9	2.3	20.0	16.0

P	GO:0009628	response to abiotic stimulus	20	9	1.7	2.6	16.1	7.3

P	GO:0009607	response to biotic stimulus	36	8	3.1	2.3	23.1	5.1

F	GO:0003824	catalytic activity	1019	253	65.2	62.6	8.8	2.2

F	GO:0005215	transporter activity	215	74	13.8	18.3	13.0	4.5

F	GO:0016787	hydrolase activity	446	125	28.6	31.0	9.5	2.7

Both P-value and Q-value for the GO-labels shown are lower than 0.05.

*TD = Tandem Duplicates; BD = Block Duplicates

As expected, and previously shown [19], tandem duplicates are enriched in genes related to pathogenesis, such as genes involved in symbiosis and genes with specific kinase activity. Moreover, 25% of all genes annotated with the GO-term "symbiosis" are part of tandem gene clusters (see Table 2). When we consider the GO-tree Cellular Component (C), we observe that tandem genes are often expressed in lysosomes, vacuoles, the external encapsulating structures, the cell wall and the extracellular region, which refers to the outermost structure of a cell (or the host cell environment in the case of an intracellular parasite).

The block duplicates are specifically enriched in the processes cell communication and signal transduction and in the functions actin binding and calcium ion binding, signal transducer activity, transcription regulator activity and receptor activity. Many of these functions, such as signal transduction and transcription but also calcium binding have been shown in several studies to be preferentially retained after a whole genome duplication because of gene dosage and gene balance effects [48-53]. Therefore, the specific retention of these genes in the small duplicated blocks in *Phytophthora *provides additional evidence for a WGD, rather than individual segmental duplications, where we would expect the opposite [51]. Additionally, the retention of calcium binding, signal transduction and cell communication proteins may also have been important in the infection process of the plant pathogen. It has been shown that the plant pathogen *Phytophthora parasitica *forms, at the site of infection, biofilms that contribute to disease development [54]. These biofilms protect the pathogen against plant defence responses and fungicidal treatments and use cell-cell communication to promote the exchange of signals and nutrients between, among others, sessile and planktonic zoospores [54]. Calcium, for example, is one of the candidate substances responsible for the chemotaxis of zoospores toward previously encysted zoospores [55-57]. Furthermore, the encystment of zoospores and the germination of cysts to form hyphae is also stimulated by nutrients and calcium (reviewed in [58]). Regarding the GO-tree cellular component, we see no preference of expression in the extracellular regions.

It is clear that both tandem and block duplicates are enriched in genes that play a role in pathogenesis. Additionally, both types of duplicates are enriched in genes that are important in the response to external, biotic and abiotic stimulus and stress. Also genes with hydrolase, transporter and catalytic activity, of which many are linked to pathogenesis, are enriched in both categories of duplicates. For example, genes of the glycosyl hydrolase family encode extracellular enzymes capable of hydrolyzing the xyloglucan component of the host cell wall, thereby facilitating the pathogen physical penetration process [59]. Although the large majority of these well-known pathogenicity genes [12,13] have clearly evolved through a continuous process of tandem duplications, we have now identified some of them as remainders of an older large-scale duplication event.

## Discussion

All three *Phytophthora *genomes contain many more small duplicated blocks than would be expected by chance alone. Furthermore, when we compare the number of duplicated blocks with those of organisms that have most probably not undergone large scale duplication events (e.g. *Drosophila melanogaster *or *Phaeodactylum tricornutum*), the difference is obvious (see Figure 3 and Table 1). Moreover, we also observed a clear difference with organisms that did undergo some segmental duplications, but no WGD. For example, *Plasmodium falciparum*, the causative agent of severe human malaria, carries multiple segmental duplications in the otherwise highly variable subtelomeres of its chromosomes [37]. However, the number of 2HOM and 3HOM blocks detected is still much smaller than in *Phytophthora*. Also in *K. lactis*, a yeast species that has not undergone a WGD, but for which eight segmental duplications have been documented, on top of some segmental duplications at the subtelomeres [39], the number of detected small duplicated blocks is much less than in *Phytophthora *[38]. On the other hand, the number of 2HOM blocks in *C. elegans*, which has undergone a few segmental duplications [36], is higher than in *P. ramorum*, but still considerably lower than in *P. infestans *and *P. sojae*. The number of 3HOM blocks on the other hand is higher than in *P. ramorum *and *P. sojae *but still lower than in *P. infestans*. However, it should be noted that the large number of 2HOM and 3HOM blocks in *C. elegans *is mainly due to a few larger segmental duplications involving between 10 and 26 genes [36]. It is also important to note that the duplicated blocks in all three *Phytophthora *species are spread over more than 60 percent of the number of scaffolds and we did not observe a bias to certain scaffolds, only a correlation between the size of the scaffold and the number of duplicated blocks, something we would expect for a WGD event. On the other hand, in *C. elegans*, 70% of the segmental duplications are intrachromosomal [36].

Because the number of blocks is directly correlated with, among other things, (i) the number of genes, (ii) the extent of genome rearrangements, and (iii) the quality of the genome assembly, we have to take these issues into account. For example, as stated before, the number of blocks in the paleopolyploid *S. cerevisiae *was lower than expected. However, this is explained by the fact that, compared to the other genomes used in this study, yeast has much fewer genes. On top of that, *S. cerevisiae *has undergone many rearrangements [28,38,60]. Figure 3B shows the percentage of 2HOM blocks for the different genomes analyzed, taking into account the number of blocks that theoretically can be found if the whole genome would have been duplicated and there would have been no genome rearrangements (translocation, loss, ...). In practice, if a complete chromosome (or scaffold in our case) with x genes has been duplicated, we would expect to find (x-1) 2HOM and (x-2) 3HOM blocks, provided none of the duplicated genes would have been translocated or lost nor other genes inserted. By dividing the number of identified blocks by the number of possible blocks, we obtain the relative number of duplicated blocks for all genomes (see Figure 3B and Table 1). Regarding 2HOM blocks, all genomes that have not undergone a large-scale duplication event, have values below 0.5%. The same is true for *Plasmodium falciparum *and *Kluyveromyces lactis*, which have only undergone some segmental duplications. For the other species, except *S. cerevisiae*, which are known to have undergone at least one genome duplication, the percentages are all > 1.5%, including *P. infestans *(2.17%) and *P. sojae *(1.91%). *P. ramorum *is just < 1.5% (1.47%), but there are no non-duplicated or segmentally duplicated genomes with a value larger than 1%. It should also be noted that, when taking the number of genes into account, the difference in the number of 2HOM blocks between *S. cerevisiae *and the non-duplicated organisms becomes larger. Also the percentage of blocks in all three *Phytophthora *species is now greater than in *C. elegans*. Moreover, the relative number of blocks in *H. sapiens *is smaller than in *P. infestans *and *P. sojae*, and similar to *P. ramorum*. Both *Tetraodon *and *Arabidopsis *still have the highest relative number of blocks.

For the 3HOM blocks, the difference between organisms that have undergone large-scale duplications and those that have not is even more pronounced (also see Table 1). For all non-duplicated genomes, the percentages are below 0.05%. When we consider the other genomes, the relative number of 3HOM blocks in *P. infestans *is the highest. For *P. sojae *and *P. ramorum *however, the percentages are lower than for *Arabidopsis *and *Tetraodon*, similar to *S. cerevisiae*, *H. sapiens *and *C. elegans*, and higher than for the other reference organisms.

**Figure 5:**
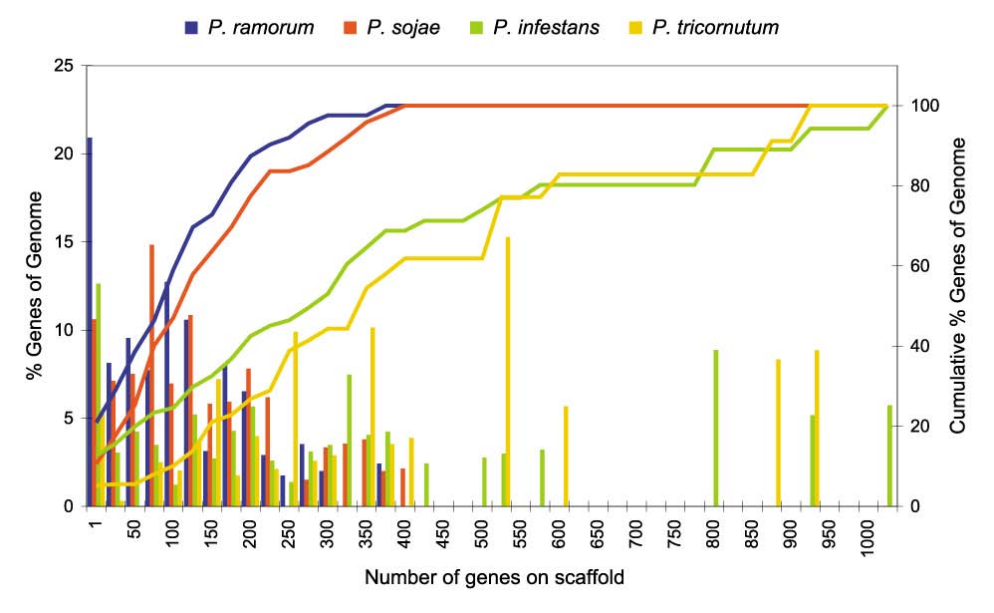
**Quality of the genome assembly for the *Phytophthora *species and for the diatom *Phaeodactylum tricornutum***. The histograms represent the percentage of genes (left Y-axis) lying on a scaffold of a certain size, measured by the number of genes on a scaffold. The corresponding lines represent the cumulative percentage of genes (right Y-axis).

It is important to realize that the genomes of all three *Phytophthora*'s still consist of scaffolds, whereas most other genomes discussed here have already been assembled into chromosomes. If the majority of scaffolds are rather small, it is obvious that it is much harder to detect large duplicated regions than for genomes that are well assembled (e.g. *Arabidopsis*, human, *Tetraodon*, *Drosophila*, ...). Therefore, we considered the distribution of scaffold sizes (i.e. number of genes on an annotated scaffold) in the different *Phytophthora *species and compared them with the diatom *Phaeodactylum tricornutum*, a genome that is also not yet assembled into chromosomes (see Figure 5). For every size bin, we counted the percentage of genes of the corresponding genome located on a scaffold of that size. In *P. ramorum *(blue histogram), we observed that the percentage of genes lying on a small scaffold (i.e. between 1 and 25 genes) is greater than for the other genomes. Also when we consider the cumulative percentage (blue line), we see that all genes (i.e. 100%) are found on scaffolds with a size smaller than 400 genes, whereas in *Phaeodactylum *we have scaffolds with more than 900 genes and in *P. infestans *with more than 1000 genes. The situation in *P. sojae *is similar to that of *P. ramorum*, although slightly better. In *Phaeodactylum*, the number of genes on small scaffolds is the lowest. Therefore because in *Phaeodactylum *the average scaffold sizes are larger, it should be easier to detect larger duplicated blocks. The fact that we could hardly find any duplicated blocks in *Phaeodactylum tricornutum *(Table 1 and Additional file 8), whereas we do find many in *P. infestans *and a considerable number in *P. ramorum *and *P. sojae *again provides support for *Phytophthora *species having undergone a large-scale or even entire genome duplication event. In addition, since the number of blocks found in the three species is linearly correlated with the assembly quality, it is likely that in *P. ramorum *and *P. sojae *the duplication signal would be more similar to *P. infestans *after improving the genome assembly.

## Conclusion

Analyses of the *Phytophthora *genomes seem to suggest that these organisms have undergone a large-scale gene duplication or WGD in their evolutionary past. Likely, this event has been shared by all three *Phytophthora *species, *P. infestans*, *P. ramorum*, and *P. sojae *and thus occurred before their speciation. Although we cannot exclude that the many small duplicated blocks have been created through many independent small block duplications, we do consider this less likely. First, when we calculate the age of the duplicated blocks a large fraction seems to have originated at the same time and they seem to be very old. If the many small blocks observed in the different *Phytophthora *genomes would have been created by a continuous mode of segmental duplications, we would expect to see an exponential decrease when plotting the age of the duplicated blocks against their frequency (i.e., many young blocks, few old ones), which is not what we observe [23,51]. It could still be that a majority of segmental duplications occurred in a short time interval in the common ancestor of all three *Phytophthora *species, but this scenario is certainly much less parsimonious than a single WGD. Furthermore, the specific enrichment of regulatory genes in the duplicated blocks provides additional support for a WGD, rather than many smaller segmental duplication events, after which where we would expect strong selection against retention of such genes [50-53,61-64].

Second, polyploids have already been identified within several species of *Phytophthora *[65-69] and other oomycetes [70] providing additional support that *P. infestans *could indeed be an ancient polyploid (with a now diploidized genome). The findings of Sansome (1977) suggested that *P. infestans *may exist in nature in the tetraploid condition and that this tetraploid might be better adapted, for instance to cooler conditions [65]. The author also claimed that the discovery of many pathogenic races of *P. infestans *[71] may be related to polyploidy in *P. infestans *[65]. The fact that we also find many genes related to pathogenesis in our set of retained duplicates might actually confirm this hypothesis.

Therefore, we conclude that *Phytophthora *is most likely an ancient polyploid. The fact that many small blocks are found suggests that its genome has been heavily rearranged following the duplication event. Furthermore, the observation that the *Phytophthora *genomes have a high repeat content, and that the gene order conservation between the genomes drops when the repeat content increases [13], further suggests that those repeats have played an important role in the rearrangement process. Haas and colleagues (2009) also suggested that the high rate of transposon activity must have occurred more recently [13], supporting our hypothesis that the WGD event has preceded the rearrangement processes. As a consequence, after tens of millions of years of evolution, and in particular for fast evolving genomes of pathogens, the paucity of a considerable number of retained homologous gene pairs in close proximity makes it almost impossible to detect statistically significant collinear regions. This might explain why no evidence has been found previously for WGD or large-scale segmental duplications in the *Phytophthora *species [12,13]. However, our newly developed strategy to look for large numbers of small duplicated blocks and compare these with genomes of other organisms for which the duplication past is better known, might still unveil ancient polyploidy events.

## Methods

### Construction of the datasets and defining homologs

The predicted protein sequences of three *Phytophthora *species, namely *Phytophthora sojae *(JGI, v1.1), *Phytophthora ramorum *(JGI, v1.1) and *Phytophthora infestans *(v1, http://www.broad.mit.edu/annotation/genome/phytophthora_infestans/) were downloaded, as well as the predicted protein sets of *Phaeodactylum tricornutum *(JGI, v1.0), *Plasmodium falciparum *(Plasmodb), *Arabidopsis thaliana *(TIGR, Release 5), *Kluyveromyces lactis *(NCBI), *Saccharomyces cerevisiae *http://www.yeastgenome.org, *Anopheles gambiae *(Ensembl, Release 52), *Caenorhabditis elegans *(Ensembl, Release 31.140), *Drosophila melanogaster *(Ensembl, Release 31.3e), *Homo sapiens *(Ensembl, Release 35) and *Tetraodon nigroviridis *(Ensembl, Release 53).

If alternative splice variants were detected for one gene, only the longest transcript was used. Also transposon-like genes were removed based on homology with known transposons retrieved from the EMBL Nucleotide Sequence Bank http://www.ebi.ac.uk/embl/ and the Swiss-Prot Protein KnowledgeBase http://www.expasy.ch/sprot/. To identify homologous genes, a similarity search was performed for every genome (BLASTP [72]; E-value cutoff E-10). Next, gene families were built with MCLBLASTLINE (Inflation Factor of 2.0; http://micans.org/mcl/, [73,74].

### Dating of paralogous and orthologous gene pairs

The fraction of synonymous substitutions per synonymous site (K_S_) is used to estimate the time of duplication or speciation between two paralogous resp. orthologous sequences. All pairwise alignments of the paralogous or orthologous nucleotide sequences belonging to a gene family were made by using CLUSTALW [75]. Gaps and adjacent divergent positions in the alignments were removed. K_S _estimates were obtained with the CODEML program [76] of the PAML package [77]. Calculations were repeated ten times to avoid incorrect K_S _estimations because of suboptimal local maxima. To exclude gene pairs that can be the result of redundancy instead of duplication, only gene pairs with a K_S _estimate higher than 0.1 were considered for further evaluation.

### Detection of (large) duplicated regions (> 2 genes)

Duplicated regions in the *Phytophthora *and reference genomes were identified with the i-ADHoRe software [24]. Homologous gene pairs, defined by MCLBLASTLINE, served as an input for the i-ADHoRe algorithm. Gene pairs of gene families with more than 100 members were omitted from the analysis. The following parameters were used: gap size of 10 genes; cluster gap of 20 genes; P-value of 0,001; Q-value of 0.9 and a minimum of three homologs to define a duplicated block.

### Detection of tandem genes and small duplicated blocks (< 4 genes)

Based on the MCLBLASTLINE-output, the order of proteins on a scaffold was converted into an order of gene families A, B, C, ..., while keeping track of the original protein IDs (see Figure 1, Panel A). Scaffolds with fewer than 6 genes were omitted from further analyses. To define all existing gene family pairs that occur next to each other in the genome, a window size of two was used to scan every scaffold. Tandem gene family pairs were excluded. Thus, in a string of, for example, A-B-C-C-B-A we define AB (BA is remapped to AB) and BC (CB is remapped to BC) as gene family pairs. CC is a tandem-pair so this pair was discarded for the block analysis and analyzed separately. With the gene pairs identified this way, we again scan every scaffold to count how many times this gene pair was found. The search is also done with a window size of 1 but when a pair is found, we move with a window size of two for the next search step only, to prevent that we would count AB two times in the example ABACD (remember that BA is remapped to AB). Therefore, when we detect AB, we jump one window further to take AC as the next pair instead of BA. When a pair is found more than once, we call it a block with two homologs (or 2HOM block). Finally, for all gene pairs that are detected more than once, a unique block ID is defined. In a post-processing step, duplicated blocks where at least one of the homologous gene pairs is a member of a large gene family (> 100 genes) were omitted from the analysis. Also duplicated blocks where one of the gene pairs has a K_S _estimate lower than 0.1 were removed to reduce the effect of redundancy. The gene IDs and coordinates of the gene pairs located in 2HOM blocks can be found in Additional file 8.

A similar strategy was applied to detect blocks with three homologous genes or 3HOM Blocks (Figure 1, Panel B). So ABC, ABB...BBBC and CBA are all remapped to ABC. However, BAC is not remapped to ABC. Also note that 2HOM blocks mean that there must be at least two successive homologs, so in the set of 3HOM blocks, the 2HOM blocks are also included. The gene IDs and coordinates of the gene pairs located in 3HOM blocks can be found in Additional file 9.

### Validation of small duplicated blocks

To examine if the number of blocks that we observe is different from what we would expect by chance only, we ran 1000 simulations for every genome. In brief, in every genome the tandem duplicates were remapped to the first gene and the gene families with more than 100 genes were removed. Next, every genome was shuffled 1000 times and each time the number of detected 2HOM and 3HOM blocks was counted. If the number of detected blocks is greater in the real data than in random data, we can conclude that the number of blocks found is significantly higher than we could expect by chance only.

### Conservation of gene orientation

For all 2HOM blocks, we compared the order of gene orientation between both homologous segments. If the gene orientation and gene order were conserved between both homologous segments (see Figure 1, Panel C) then we concluded that the orientation is conserved. If the gene order is inverted together with the orientation (see Figure 1, Panel D), then we also conclude that the orientation in this block is conserved. In all other cases, we consider the orientation as not conserved. The same analysis was done on all randomized (shuffled) genomes created for the block detection strategy.

### GO annotation and GO enrichment

The proteins of all *Phytophthora *genomes were annotated using Gene Ontology (GO) [78]. In a first step, all genes were annotated for protein function using InterProScan [79]. Next, the resulting InterPro annotation was converted into GO annotation. Proteins mapped to a particular GO category were also explicitly included into all parental categories. All GO categories were also mapped into the GO Slim categories. The statistical significance of functional GO Slim enrichment was evaluated by using the hypergeometric distribution, whereas multiple hypotheses testing was done by using FDR [80].

## Authors' contributions

CM designed the research, analyzed the data and wrote the paper. YVdP designed the research, supervised the project and wrote the paper.

## Supplementary Material

Additional file 1 **Detection of 2HOM blocks in the *Phytophthora *and reference genomes**. The real data are represented by the pink triangle data points, while the 1000 simulations or random data is represented by blue curves. The number of observations in the real data is always equal to 1 since there is only one real dataset per genome, while there are 1000 random datasets per genome. Click here for file

Additional file 2 **Detection of 3HOM blocks in the *Phytophthora *and reference genomes**. Interpretation is as in Additional file 1. Click here for file

Additional file 3 **Frequency of scaffolds with a certain number of duplicated blocks**. Click here for file

Additional file 4 **Relation between the size of a scaffold and the number of detected blocks**. The graphs with the yellow bars show the relation between the number of detected 2HOM blocks and the average scaffold size for all three *Phytophthora *species. Click here for file

Additional file 5 **Supporting methods and results**. This file contains additional information about the applied methods and the results regarding the functional clustering analysis. Click here for file

Additional file 6 **Intergenic distances in the duplicated blocks**. Comparison of the Intergenic Distances in 2HOM Blocks (pink), 3HOM Blocks (green) and the whole proteome (blue) in the *Phytophthora *and reference genomes. Click here for file

Additional file 7 **Orientation conservation in the 2HOM and 3HOM blocks of the *Phytophthora *and reference genomes**. Interpretation is as in Additional file 1. Click here for file

Additional file 8 **Gene IDs and gene coordinates of the *Phytophthora *duplicates located in 2HOM blocks**. Click here for file

Additional File 9 **GeneIDs and gene coordinates of the *Phytophthora *duplicates located in 3HOM blocks**. Click here for file
