# Supplementary figures and images for: The hidden duplication past of the plant pathogen Phytophthora and its consequences for infection
Source: BMC Genomics. 2010 Jun 3;11:353. doi: 10.1186/1471-2164-11-353 (PMC2996974; doi:10.1186/1471-2164-11-353)

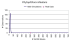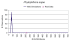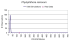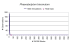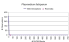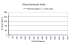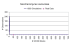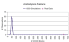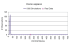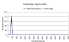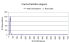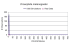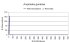

Supplement: Additional file 1 — Detection of 2HOM blocks in the Phytophthora and reference genomes. The real data are represented by the pink triangle data points, while the 1000 simulations or random data is represented by blue curves. The number of observations in the real data is always equal to 1 since there is only one real dataset per genome, while there are 1000 random datasets per genome. [file 1471-2164-11-353-S1.PDF]

### *Phytophthora infestans*

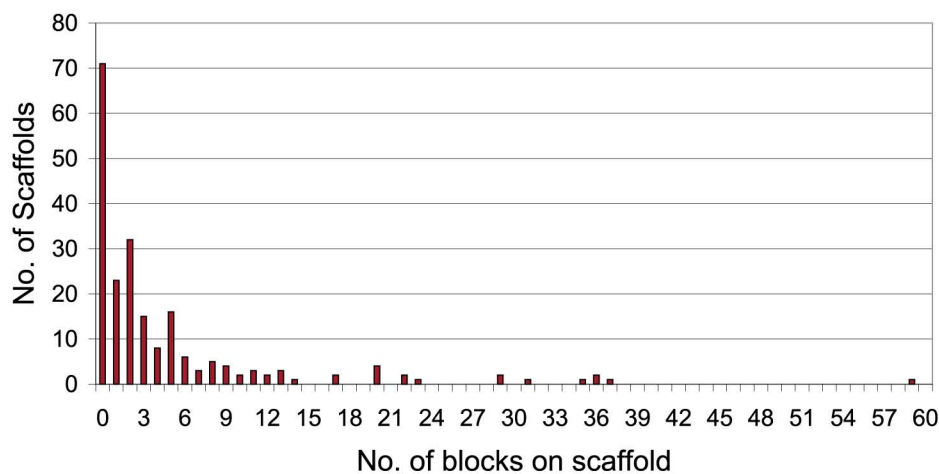

### *Phytophthora sojae*

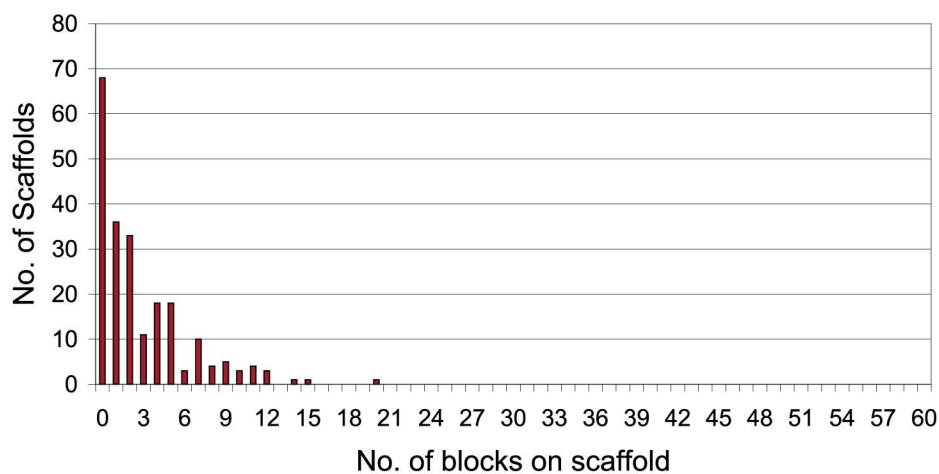

### *Phytophthora ramorum*

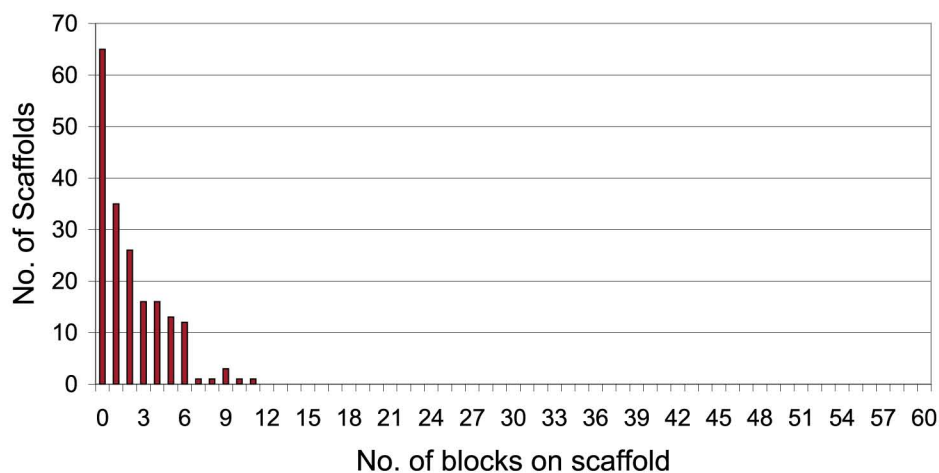

Supplement: Additional file 3 — Frequency of scaffolds with a certain number of duplicated blocks. [file 1471-2164-11-353-S3.PDF]

### *Phytophthora infestans*

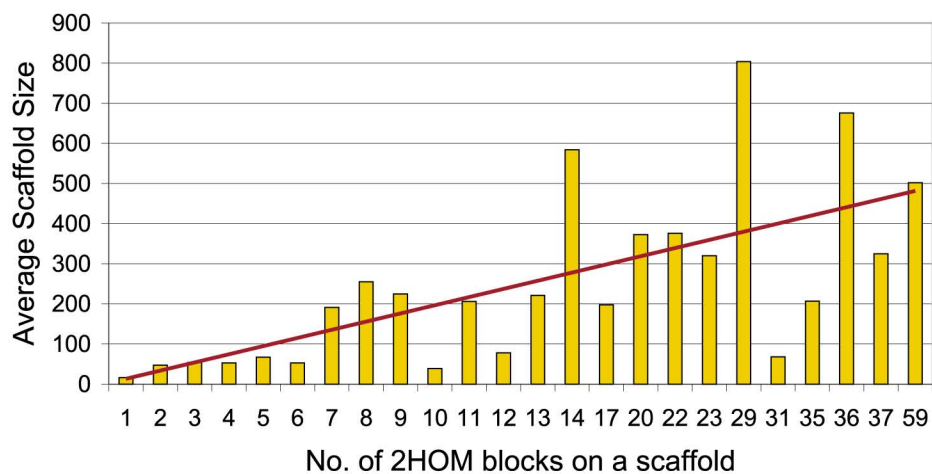

### *Phytophthora sojae*

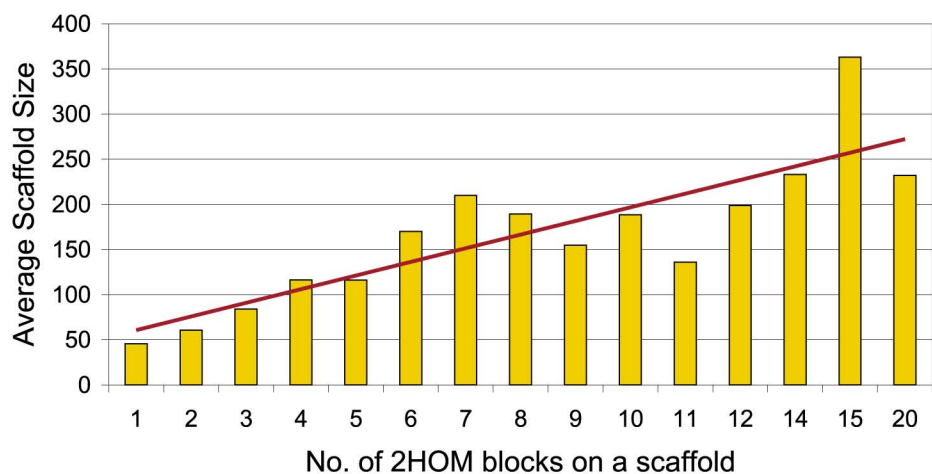

### *Phytophthora ramorum*

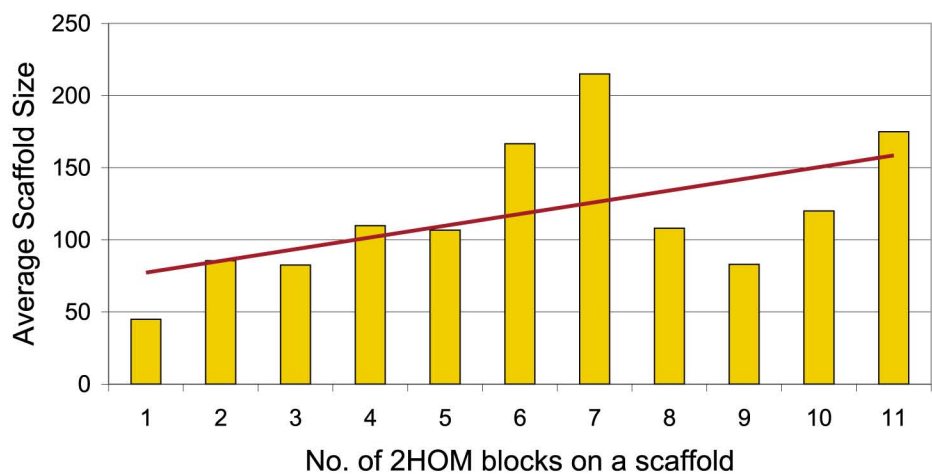

Supplement: Additional file 4 — Relation between the size of a scaffold and the number of detected blocks. The graphs with the yellow bars show the relation between the number of detected 2HOM blocks and the average scaffold size for all three Phytophthora species. [file 1471-2164-11-353-S4.PDF]
