# Supplementary material for: The hidden duplication past of the plant pathogen Phytophthora and its consequences for infection
Source: BMC Genomics. 2010 Jun 3;11:353. doi: 10.1186/1471-2164-11-353 (PMC2996974; doi:10.1186/1471-2164-11-353)
Supplement: Additional file 2 — Detection of 3HOM blocks in the Phytophthora and reference genomes. Interpretation is as in Additional file 1. [file 1471-2164-11-353-S2.PDF]

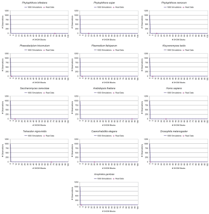
Phytophthora infestans
Phytophthora sojae
Phytophthora ramorum
Phaeodactylum tricornutum
Plasmodium falciparum
Kluyveromyces lactis
Saccharomyces cerevisiae
Arabidopsis thaliana
Homo sapiens
Tetraodon nigroviridis
Caenorhabditis elegans
Drosophila melanogaster
Anopheles gambiae
1000 Simulations
Real Data
