# Supplementary material for: The hidden duplication past of the plant pathogen Phytophthora and its consequences for infection
Source: BMC Genomics. 2010 Jun 3;11:353. doi: 10.1186/1471-2164-11-353 (PMC2996974; doi:10.1186/1471-2164-11-353)
Supplement: Additional file 5 — Supporting methods and results. This file contains additional information about the applied methods and the results regarding the functional clustering analysis. [file 1471-2164-11-353-S5.DOC]

# Supporting Methods and Results

# The hidden duplication past of the plant pathogen *Phytophthora* and its consequences for infection

# Cindy Martens^1,2^ and Yves Van de Peer^1,2§^

^1^ Department of Plant Systems Biology, VIB, Technologiepark 927, B-9052 Ghent, Belgium

^2^ Bioinformatics and Evolutionary Genomics, Department of Molecular Genetics,
Technologiepark 927, Ghent University, B-9052 Ghent, Belgium

^§^ Corresponding author

Email addresses:

C.M.: [cindy.martens@psb.vib-ugent.be](mailto:cindy.martens@psb.vib-ugent.be)

Y.V.d.P.: [yves.vandepeer@psb.vib-ugent.be](mailto:yves.vandepeer@psb.vib-ugent.be)

## Methods

**Functional clustering**

For the detection of clusters of functionally related genes, the program C-Hunter was applied [1]. C-Hunter is a clustering algorithm which incorporates the knowledge of gene function derived from Gene Ontology with the organization of genes on chromosomes. In order to use C-Hunter; we supplied the program with our own data sets.

## Results

**Real blocks or functional clustering?**

Our results suggest that the small duplicated blocks we identify in *Phytopthora* genomes have been created through a large-scale or entire genome duplication event. The fact that the blocks are small suggests an ancient duplication followed by many genome rearrangements. As previously mentioned, it has been shown that the *Phytophthora* genomes are subject to genome rearrangements and we assume that the high number of transposons is partly mediating this process [2-7]. However, the occurrence of duplicated operon-like structures or functional clusters could be an alternative explanation. Although operons represent a common form of gene organization in bacteria and archaea, polycistronic transcription in eukaryotes appears to be limited to very few phyla. In flies [8], mammals [9] and plants [10, 11] only a few cases are known, but in trypanosomes [12] and nematodes [13-16], the operon phenomenon is common. In most bacterial operons, the genes have a related function. For example Salgaso and colleagues showed that ~80% of adjacent gene pairs within *E. coli* operons belong to the same functional class [17]. Where in bacteria we can explain the existence of operons by their crucial importance in bacterial speciation through horizontal transfer [18], in eukaryotes we need another viable explanation. Since polycistronic transcription can eliminate the need for individual promoter elements, operons could serve to reduce the genome size, which can be interesting for genomes with a fast generation time [16].

To be able to detect functional clusters, the proteins of all *Phytophthora* genomes were functionally annotated using Gene Ontology (see Methods). Next, the software C-Hunter was applied to identify gene clusters in eukaryotic genomes using the functional categories defined in graph-based vocabularies such as Gene Ontology (GO) [1]. Therefore the genes in such clusters only have to share a common function, but do not require, for example, similar expression patterns. Functional clusters were found in the different genomes, but almost all of them are just tandem arrays. Some non-tandem clusters were also detected, but none of them were overlapping with the previously detected duplicated blocks. However, when applying C-Hunter, we have to take into account the quality of the GO-annotation. If there are many genes that are unknown, it becomes very difficult to detect functional clusters. In *Phytophthora infestans*, *Phytophthora sojae* and *Phytophthora ramorum* only 42%, respectively 38% and 43% of the genes have been annotated with a GO-label (cfr. 51% in *C. elegans*). Therefore, we also used another strategy to examine whether the small duplicated blocks could be operons.

Since operons are polycistronic transcription units, i.e. they have only one promoter upstream of the cluster, and as a result, the intergenic space in the cluster is reduced. If the intergenics in the small duplicated blocks would be smaller than in the rest of the proteome, we could hypothesize that those genes are regulated by a shared promoter at the beginning of the cluster (or block). Therefore, we plotted the length of the intergenics for three different categories: (i) the intergenics in 2HOM blocks, (ii) in 3HOM blocks, (iii) in the whole genome. This was done for all genomes in our dataset; including the reference genomes (see Figure S5). As can be seen in this figure, we do not see much difference, even not for *C. elegans*. This means that in *C. elegans* the small blocks are not biased to the operons either. Taken into account that in the *Phytophthora* species we found no functional clustering within the small duplicated blocks, nor found evidence for shorter intergenic distance, we see no reason to assume that those small blocks are duplicated operons. Moreover, since the size of all *Phytophthora* genomes is much larger than other plant pathogens, the explanation that operons in eukaryotes serve to reduce the genome size is also not applicable.

## References

1. Yi G, Sze SH, Thon MR: **Identifying clusters of functionally related genes in genomes**. *Bioinformatics (Oxford, England)* 2007, **23**(9):1053-1060.

2. Tyler BM, Tripathy S, Zhang X, Dehal P, Jiang RH, Aerts A, Arredondo FD, Baxter L, Bensasson D, Beynon JL *et al*: **Phytophthora genome sequences uncover evolutionary origins and mechanisms of pathogenesis**. *Science (New York, NY* 2006, **313**(5791):1261-1266.

3. Haas BJ, Kamoun S, Zody MC, Jiang RH, Handsaker RE, Cano LM, Grabherr M, Kodira CD, Raffaele S, Torto-Alalibo T *et al*: **Genome sequence and analysis of the Irish potato famine pathogen Phytophthora infestans**. *Nature* 2009.

4. Jiang RH, Govers F: **Nonneutral GC3 and retroelement codon mimicry in Phytophthora**. *Journal of molecular evolution* 2006, **63**(4):458-472.

5. Judelson HS: **Sequence variation and genomic amplification of a family of Gypsy-like elements in the oomycete genus Phytophthora**. *Molecular biology and evolution* 2002, **19**(8):1313-1322.

6. Ah Fong AM, Judelson HS: **The hAT -like DNA transposon DodoPi resides in a cluster of retro- and DNA transposons in the stramenopile Phytophthora infestans**. *Molecular genetics and genomics* 2004, **271**(5):577-585.

7. Jiang RH, Dawe AL, Weide R, van Staveren M, Peters S, Nuss DL, Govers F: **Elicitin genes in Phytophthora infestans are clustered and interspersed with various transposon-like elements**. *Molecular genetics and genomics* 2005, **273**(1):20-32.

8. Andrews J, Smith M, Merakovsky J, Coulson M, Hannan F, Kelly LE: **The stoned locus of Drosophila melanogaster produces a dicistronic transcript and encodes two distinct polypeptides**. *Genetics* 1996, **143**(4):1699-1711.

9. Lee SJ: **Expression of growth/differentiation factor 1 in the nervous system: conservation of a bicistronic structure**. *Proc Natl Acad Sci U S A* 1991, **88**(10):4250-4254.

10. Blumenthal T: **Gene clusters and polycistronic transcription in eukaryotes**. *Bioessays* 1998, **20**(6):480-487.

11. Garcia-Rios M, Fujita T, LaRosa PC, Locy RD, Clithero JM, Bressan RA, Csonka LN: **Cloning of a polycistronic cDNA from tomato encoding gamma-glutamyl kinase and gamma-glutamyl phosphate reductase**. *Proc Natl Acad Sci U S A* 1997, **94**(15):8249-8254.

12. Vanhamme L, Pays E: **Control of gene expression in trypanosomes**. *Microbiol Rev* 1995, **59**(2):223-240.

13. Zorio DA, Cheng NN, Blumenthal T, Spieth J: **Operons as a common form of chromosomal organization in C. elegans**. *Nature* 1994, **372**(6503):270-272.

14. Blumenthal T: **Trans-splicing and polycistronic transcription in Caenorhabditis elegans**. *Trends Genet* 1995, **11**(4):132-136.

15. Blumenthal T, Gleason KS: **Caenorhabditis elegans operons: form and function**. *Nat Rev Microbiol* 2003, **4**(2):112-120.

16. Nimmo R, Woollard A: **Widespread organisation of C. elegans genes into operons: fact or function?** *Bioessays* 2002, **24**(11):983-987.

17. Salgado H, Moreno-Hagelsieb G, Smith TF, Collado-Vides J: **Operons in Escherichia coli: genomic analyses and predictions**. *Proc Natl Acad Sci U S A* 2000, **97**(12):6652-6657.

18. Lawrence JG: **Selfish operons and speciation by gene transfer**. *Trends Microbiol* 1997, **5**(9):355-359.
