# Supplementary material for: The hidden duplication past of the plant pathogen Phytophthora and its consequences for infection
Source: BMC Genomics. 2010 Jun 3;11:353. doi: 10.1186/1471-2164-11-353 (PMC2996974; doi:10.1186/1471-2164-11-353)
Supplement: Additional file 6 — Intergenic distances in the duplicated blocks. Comparison of the Intergenic Distances in 2HOM Blocks (pink), 3HOM Blocks (green) and the whole proteome (blue) in the Phytophthora and reference genomes. [file 1471-2164-11-353-S6.PDF]

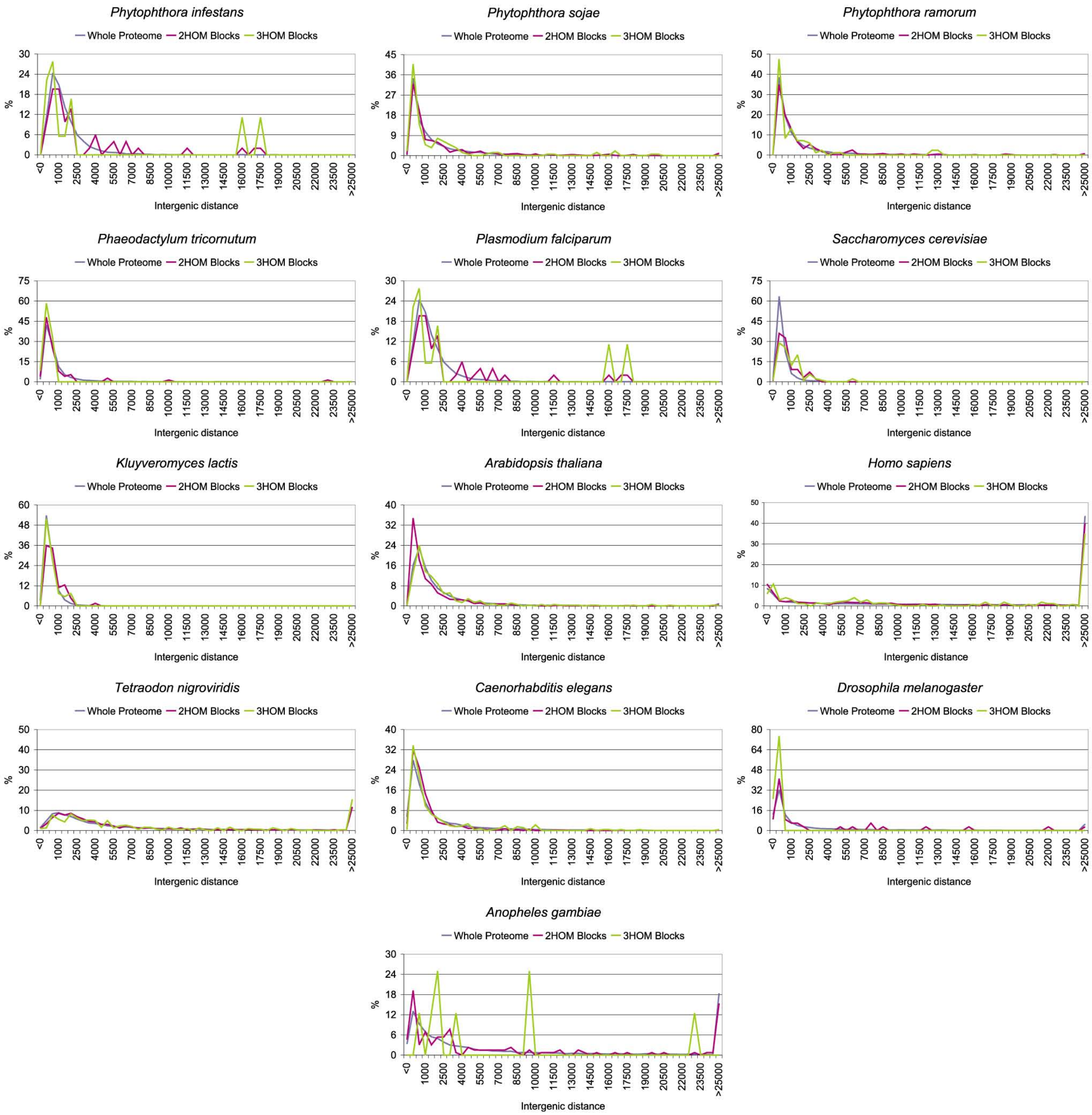

Phytophthora infestans
Whole Proteome 2HOM Blocks 3HOM Blocks
%
0 6 12 18 24 30
<0 1000 2500 4000 5500 7000 8500 10000 11500 13000 14500 16000 17500 19000 20500 22000 23500 >25000
Intergenic distance
Phytophthora sojae
Whole Proteome 2HOM Blocks 3HOM Blocks
%
0 9 18 27 36 45
<0 1000 2500 4000 5500 7000 8500 10000 11500 13000 14500 16000 17500 19000 20500 22000 23500 >25000
Intergenic distance
Phytophthora ramorum
Whole Proteome 2HOM Blocks 3HOM Blocks
%
0 10 20 30 40 50
<0 1000 2500 4000 5500 7000 8500 10000 11500 13000 14500 16000 17500 19000 20500 22000 23500 >25000
Intergenic distance
Phaeodactylum tricornutum
Whole Proteome 2HOM Blocks 3HOM Blocks
%
0 15 30 45 60 75
<0 1000 2500 4000 5500 7000 8500 10000 11500 13000 14500 16000 17500 19000 20500 22000 23500 >25000
Intergenic distance
Plasmodium falciparum
Whole Proteome 2HOM Blocks 3HOM Blocks
%
0 6 12 18 24 30
<0 1000 2500 4000 5500 7000 8500 10000 11500 13000 14500 16000 17500 19000 20500 22000 23500 >25000
Intergenic distance
Saccharomyces cerevisiae
Whole Proteome 2HOM Blocks 3HOM Blocks
%
0 15 30 45 60 75
<0 1000 2500 4000 5500 7000 8500 10000 11500 13000 14500 16000 17500 19000 20500 22000 23500 >25000
Intergenic distance
Kluyveromyces lactis
Whole Proteome 2HOM Blocks 3HOM Blocks
%
0 12 24 36 48 60
<0 1000 2500 4000 5500 7000 8500 10000 11500 13000 14500 16000 17500 19000 20500 22000 23500 >25000
Intergenic distance
Arabidopsis thaliana
Whole Proteome 2HOM Blocks 3HOM Blocks
%
0 8 16 24 32 40
<0 1000 2500 4000 5500 7000 8500 10000 11500 13000 14500 16000 17500 19000 20500 22000 23500 >25000
Intergenic distance
Homo sapiens
Whole Proteome 2HOM Blocks 3HOM Blocks
%
0 10 20 30 40 50
<0 1000 2500 4000 5500 7000 8500 10000 11500 13000 14500 16000 17500 19000 20500 22000 23500 >25000
Intergenic distance
Tetraodon nigroviridis
Whole Proteome 2HOM Blocks 3HOM Blocks
%
0 10 20 30 40 50
<0 1000 2500 4000 5500 7000 8500 10000 11500 13000 14500 16000 17500 19000 20500 22000 23500 >25000
Intergenic distance
Caenorhabditis elegans
Whole Proteome 2HOM Blocks 3HOM Blocks
%
0 8 16 24 32 40
<0 1000 2500 4000 5500 7000 8500 10000 11500 13000 14500 16000 17500 19000 20500 22000 23500 >25000
Intergenic distance
Drosophila melanogaster
Whole Proteome 2HOM Blocks 3HOM Blocks
%
0 16 32 48 64 80
<0 1000 2500 4000 5500 7000 8500 10000 11500 13000 14500 16000 17500 19000 20500 22000 23500 >25000
Intergenic distance
Anopheles gambiae
Whole Proteome 2HOM Blocks 3HOM Blocks
%
0 6 12 18 24 30
<0 1000 2500 4000 5500 7000 8500 10000 11500 13000 14500 16000 17500 19000 20500 22000 23500 >25000
Intergenic distance
