# Supplementary material for: The hidden duplication past of the plant pathogen Phytophthora and its consequences for infection
Source: BMC Genomics. 2010 Jun 3;11:353. doi: 10.1186/1471-2164-11-353 (PMC2996974; doi:10.1186/1471-2164-11-353)
Supplement: Additional file 7 — Orientation conservation in the 2HOM and 3HOM blocks of the Phytophthora and reference genomes. Interpretation is as in Additional file 1. [file 1471-2164-11-353-S7.PDF]

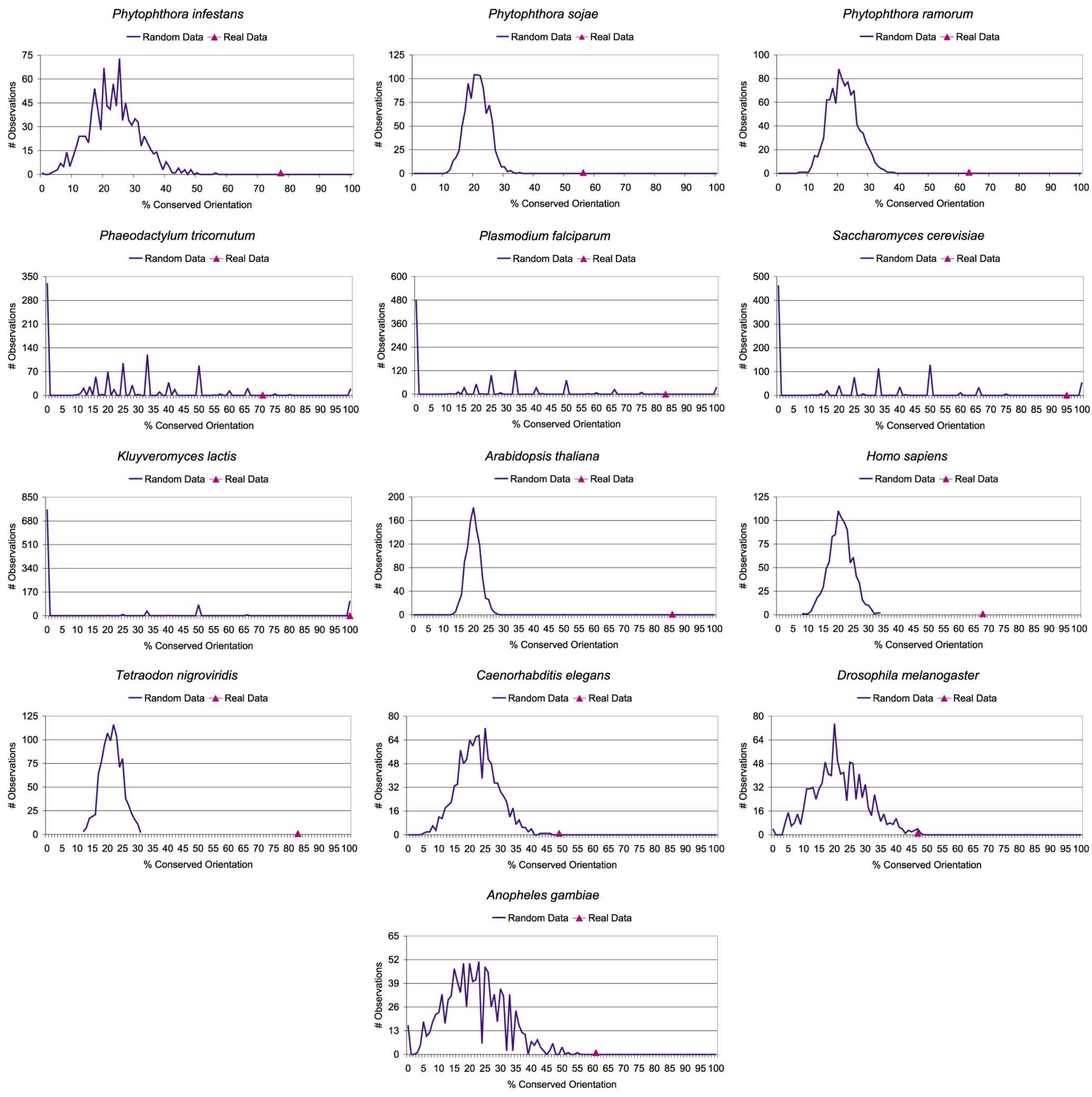

Phytophthora infestans
Random Data
Real Data
Observations
% Conserved Orientation
Phytophthora sojae
Random Data
Real Data
Observations
% Conserved Orientation
Phytophthora ramorum
Random Data
Real Data
Observations
% Conserved Orientation
Phaeodactylum tricornutum
Random Data
Real Data
Observations
% Conserved Orientation
Plasmodium falciparum
Random Data
Real Data
Observations
% Conserved Orientation
Saccharomyces cerevisiae
Random Data
Real Data
Observations
% Conserved Orientation
Kluyveromyces lactis
Random Data
Real Data
Observations
% Conserved Orientation
Arabidopsis thaliana
Random Data
Real Data
Observations
% Conserved Orientation
Homo sapiens
Random Data
Real Data
Observations
% Conserved Orientation
Tetraodon nigroviridis
Random Data
Real Data
Observations
% Conserved Orientation
Caenorhabditis elegans
Random Data
Real Data
Observations
% Conserved Orientation
Drosophila melanogaster
Random Data
Real Data
Observations
% Conserved Orientation
Anopheles gambiae
Random Data
Real Data
Observations
% Conserved Orientation
